# Can pre-trained convolutional neural networks be directly used as a feature extractor for video-based neonatal sleep and wake classification?

**DOI:** 10.1186/s13104-020-05343-4

**Published:** 2020-11-04

**Authors:** Muhammad Awais, Xi Long, Bin Yin, Chen Chen, Saeed Akbarzadeh, Saadullah Farooq Abbasi, Muhammad Irfan, Chunmei Lu, Xinhua Wang, Laishuan Wang, Wei Chen

**Affiliations:** 1 Center for Intelligent Medical Electronics, Department of Electronic Engineering, School of Information Science and Technology, Fudan University, Shanghai, 200433 China; 2 Department of Electrical Engineering, Eindhoven University of Technology, Den Dolech 2, 5612 AZ Eindhoven, The Netherlands; 3 Connected Care and Personal Health Department, Philips Research, Shanghai, China; 4 Department of Neonatology, Children’s Hospital of Fudan University, Shanghai, 200032 China; 5 Department of Neurology, Children’s Hospital of Fudan University, Shanghai, 200032 China; 6 China, and Human Phenome Institute Fudan University, Shanghai, China; 7 Department of Family Care Solutions, Philips Research, Eindhoven, 5656 AE The Netherlands

**Keywords:** Convolutional neural networks (CNNs), Video electroencephalogram (VEEG), Neonatal sleep, Sleep and wake classification, Feature extraction

## Abstract

**Objective:**

In this paper, we propose to evaluate the use of pre-trained convolutional neural networks (CNNs) as a features extractor followed by the Principal Component Analysis (PCA) to find the best discriminant features to perform classification using support vector machine (SVM) algorithm for neonatal sleep and wake states using Fluke^®^ facial video frames. Using pre-trained CNNs as a feature extractor would hugely reduce the effort of collecting new neonatal data for training a neural network which could be computationally expensive. The features are extracted after fully connected layers (FCL’s), where we compare several pre-trained CNNs, e.g., VGG16, VGG19, InceptionV3, GoogLeNet, ResNet, and AlexNet.

**Results:**

From around 2-h Fluke^®^ video recording of seven neonates, we achieved a modest classification performance with an accuracy, sensitivity, and specificity of 65.3%, 69.8%, 61.0%, respectively with AlexNet using Fluke^®^ (RGB) video frames. This indicates that using a pre-trained model as a feature extractor could not fully suffice for highly reliable sleep and wake classification in neonates. Therefore, in future work a dedicated neural network trained on neonatal data or a transfer learning approach is required.

## Introduction

Sleep is an essential behavior for the development of the nervous system in neonates [1–3]. Normally, newborn babies sleep between 16 and 18 h per day. Continuous sleep tracking and assessment could potentially provide an indicator of brain development over time [4, 5]. To achieve this, automatic sleep and wake analysis is required, which can offer valuable information on a neonate’s mental and physical growth, not only for healthcare professionals but also for parents [6].

Currently, Video electroencephalogram (VEEG) is considered as a gold standard for neonatal sleep monitoring, which requires a number of sensors and electrodes attached to a neonate’s skin to collect multiple-channel EEG signals [7–9]. In addition, the use of VEEG is labor-intensive, where the human effort on annotating sleep states is required [10]. Therefore, one would demand a contact-free sleep monitoring system for neonates. In recent years, unobtrusive or contact-free approaches have gained a lot of attention for sleep monitoring [11–16]. All these methods are more successful in adults [17, 18]. In contrast, video-based methods appear to be a promising approach since it is more comfortable and convenient to use both in the home or in the hospitals [19, 20]. With the advancements in deep learning algorithms and clinical research on neonatal facial patterns [21, 22], a new, unobtrusive approach of monitoring sleep patterns has been proposed [23, 24]. However, evaluation of the deep learning models demands big database to train the prediction model.

The main contributions of this work include: (a) extracting features from well-known CNNs, e.g., VGG-16, VGG-19, InceptionV3, GoogLeNet, ResNet, and AlexNet, (b) comparing different color palette (amber, high contrast, red-blue, hot metal, and grayscale) from RGB and thermal video frames, and (c) evaluating the extracted features using Principal Component Analysis (PCA) followed by Support Vector Machine (SVM) to classify neonatal sleep and wake classification. As this was an explorative study, to evaluate the feasibility of a pre-trained model as a feature extractor to classify neonates’ sleep and wake states using video frames, we started with a small pilot study population of neonate’s video frames data by adopting a robust and less computational complex approach to classify sleep states.

## Main text

### Subject database

Video and VEEG data from seven neonates were collected retrospectively by a pediatrician at the Children’s Hospital affiliated to Fudan University, Shanghai, China [25]. The detailed descriptions of the demographics and physical conditions of the neonates are shown in Additional file 1: Table S1. Annotation of sleep and wake states was performed by a professional neurologist on each 30-sec VEEG epoch and video frames, respectively, according to the American Academy of Sleep Medicine (AASM) [26].

### Intensity-based detection

**Fig. 1:**
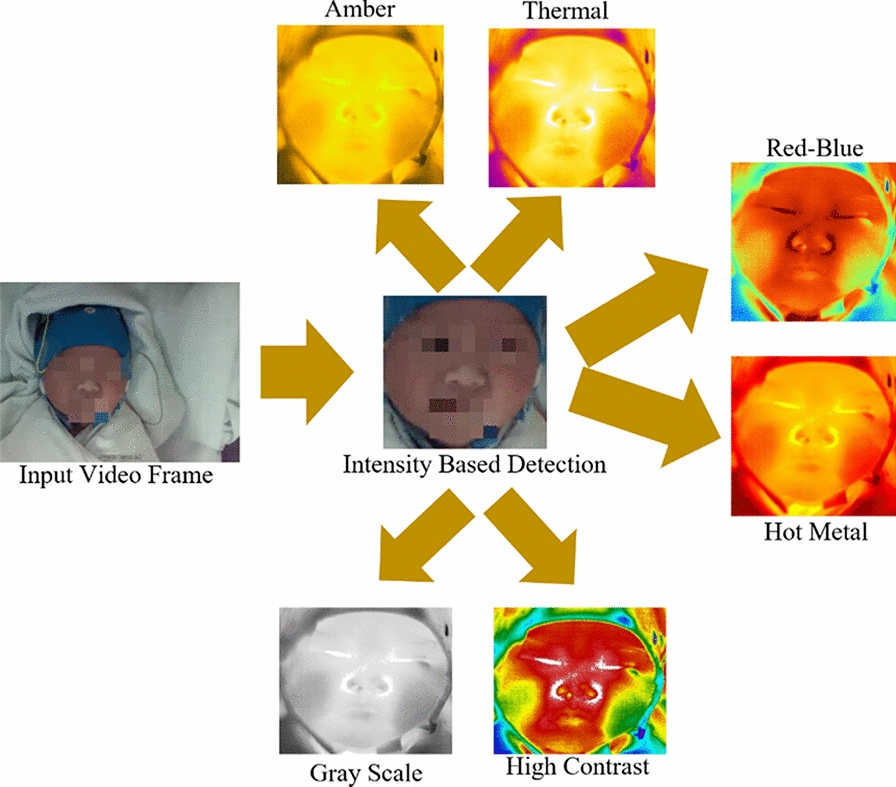
Neonatal face detection using the intensity-based detection method

To enable identifying sleep and wake states for neonates using video frames, it is required to have precise face detection in Fluke^®^ video [27]. Detail description of Intensity-based detection has been discussed in our previous paper [25]. Figure 1 shows the input video frame, and the neonatal facial region is detected using an intensity-based method. After that, the detected RGB facial region is mapped on other color palettes (thermal) of the video frames to extract the facial region.

### Pre-trained CNN models

Our proposed method is to classify neonatal sleep and wake states using pre-trained CNNs. Usually, initial layers of CNNs capture basic input image features like spots, boundaries, and colors pattern that are inattentive by the deeper hidden layers to form complex higher-level feature patterns to present a better-off image illustration [28]. Each layer of the CNNs output acts as an activation unit for the input images. Literature studies reveal that while using pre-trained CNNs for feature extraction, the features are usually extracted from the fully-connected layers (FCL’s) right before the final output classification layer [29, 30]. Considering this motivation, we extracted the features from from the FCL’s of a pre-trained network. The detailed descriptions of all the pre-trained models is mentioned in Additional file 2: Table S2. In the following, we briefly introduce the existing pre-trained models as well as PCA and SVM used in our work.

*VGG16 and VGG19 Model* VGG model [31] contains a stack of convolutional layers followed by three FCL’s. In this work, we used both pre-trained VGG16 and VGG19 models, and features were extracted from the last three FCLs.

*AlexNet architecture* The architecture of AlexNet [32] that contains a total of eight layers. In this work, we extracted features from the last two FCL’s of the pre-trained AlexNet.

*ResNet-18* The baseline structure of the residual network (ResNet) [33] is the same as the other CNNs, except that a shortcut link is added to each pair of 3 × 3 filters. To classify a neonate's sleep and wake states, we extract 1000 features from the last FCL of the pre-trained ResNet-18 model.

*GoogLeNet* GoogLeNet [34] has unique features that help them to achieve state-of-the-art results and outperform other previous networks, e.g., 1 × 1 convolution is used as a dimension reduction to reduce computation usage. In this work, we have used the pre-trained GoogLeNet network, and features are extracted from the last “FC1000” layer.

*InceptionV3* Inception-V3 is the factorization idea in the third iteration of GoogLeNet [35]. The last FCL is used to extract the features from the pre-trained Inception–V3 model to perform neonatal sleep and wake classification.

*Principal component analysis (PCA)* PCA is a method to differentiate the discriminant features in the dataset by suppressing variations [36]. In this paper, once the features are extracted from FCL’s of CNNs, we input these features to PCA to find the best-discriminated features, to help SVM to classify neonates sleep and wake states at the next stage.

*Support vector machine (SVM)* Based on features extracted from the pre-trained CNNs, we employed an SVM classifier to classify neonatal sleep and wake states [37, 38]. We have used the “classificationLearner“ app in Matlab R2018b with the SVM default setting (kernel function = ‘linear,’ box constraint = 1) to perform the classification.

### Results and discussion

Twenty-two experiments were conducted on RGB and thermal videos, respectively. For evaluation purposes, all the results are expressed in terms of sensitivity(Se), specificity(Sp), precision(p), and accuracy(Ac), obtained using five-fold cross-validation. The results are validated with the VEEG annotations.

**Fig. 2:**
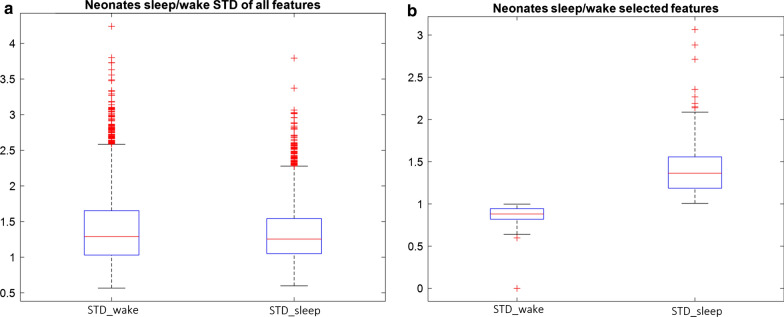
**a** STD of all features extracted from the pre-trained AlexNet (FCL7). **b** STD of discriminant features after PCA extracted from the pre-trained AlexNet (FCL7). The central redline (inside the blue boxes) indicates the median, and the bottom and top edges of the blue-box indicate the 25th and 75th percentiles of data points, respectively. The whiskers extend to the most extreme data points not considered outliers, and the outliers are plotted individually using the ‘ + ’ symbol

Table 1 shows the sleep and wake classification results obtained by the SVM classifier after feature extraction using different pre-trained CNNs. We observed that the overall performance of using FCL6-7-8 in VGG-16 and VGG19, FCL8 in AlexNet, and FCL in inceptionV3, ResNet-18, and GoogLeNet was low when used to classify neonatal sleep and wake states. Multifarious statistical results obtained via SVM to classify neonatal sleep and wake states show a disproportionate pattern. However, RGB-InceptionV3 (FCL) shows the best values for Se is 97.4%, but Ac drops to 55.1%, and similarly RGB-VGG16 (FCL8) and RGB-VGG19 (FCL8) shows Se of 90.0%, but overall accuracy is drops to 66.2% and 65.2% respectively. However, features extracted from AlexNet (FCL7) trained via SVM shows the best optimal results with an Ac of 65.3%, Se 69.8%, and Sp of 61.0% to classify neonatal sleep and wake states. In contract to the other features extracted values from pre-trained networks, features extracted from AlexNet (FCL7) contains discriminant features that assist SVM to classify neonate’s sleep and wake stage. One of the main reason to achieved higher statistical results using pre-trained AlexNet is that as pre-trained, AlexNet was originally trained on just over a million images as compared to other CNNs that were trained on more the 15 million images, depicting more complex features architecture values at different FCL’s [31, 39]. It is observed that in AlexNet, the first layer has a filter of size 11 × 11, and the second layer has a 5x5 filter, and so on, there is no standard about filter sizes and max pooling. The convolutions for each layer are decided purely experimentally. In contrary to that, other CNNs have standard protocol such as in VGG-Net, all the convolution kernels are of size 3x3, and max-pooling is done after 2 or 3 layers of convolutions. GoogLeNet works on a parallel combination of 1x1, 3x3, and 5x5 convolutional filters. The overall complex nature of pre-trained CNNs distinguished AlexNet to obtain better performance to classify neonatal sleep and wake states. Figure 2a shows the standard deviation (STD) of all the sleep and wake features extracted from AlexNet FCL7. It is observed that most of the sleep and wake extracted features from FCL7 are lies almost in the same region. However, AlexNet shows slightly better performance than other extracted features using SVM; one of the main reasons is that the corresponding trained features are quite separated from each other. Figure 2b depicts the STD of discriminant corresponding features extracted after PCA from pre-trained AlexNet (FCL7). These discriminant AlexNet (FCL7) features help to achieve better neonatal sleep and wake classification accuracy as compared to other pre-trained CNNs.

**Table 1:** Neonatal sleep and wake classification results (five-fold cross-validation) using different pre-trained CNNs combined with an SVM classifier

Video frame			Se %	Sp %	Ac %	P %
			SVM			
RGB	VGG16	FCL6	52.4	64.05	58.3	57.7
		FCL7	52.6	75.1	64.3	66.5
		FCL8	40.9	*90.0*	66.2	40.2
Thermal		FCL6	73.1	40.16	58.4	60.2
		FCL7	72.4	40.3	58.1	60.0
		FCL8	71.2	41.5	58.0	60.1
RGB	AlexNet	FCL7	*69.8*	*61.0*	*65.3*	*62.7*
Thermal		FCL7	61.0	40.4	49.9	55.0
RGB		FCL8	59.5	71.2	65.7	64.7
Thermal		FCL8	56.5	60.0	58.4	52.7
RGB	VGG19	FCL6	81.9	36.4	52.6	54.6
		FCL7	81.0	36.1	51.9	54.5
		FCL8	40.9	*90.0*	65.2	*79.4*
Thermal		FCL6	63.6	54.2	57.1	63.2
		FCL7	67.6	45.0	54.6	60.3
		FCL8	62.1	52.4	58.1	61.8
RGB	InceptionV3	FCL	*97.4*	30.0	55.1	52.5
Thermal		FCL	67.3	47.6	58.7	61.4
RGB	ResNet-18	FCL	73.2	50.8	61.2	58.2
Thermal		FCL	66.7	46.7	58.3	60.8
RGB	GoogLeNet	FCL	77.7	41.8	55.3	55.5
Thermal		FCL	66.6	46.7	57.83	60.8

*^true positive (TP) = VEEG depict sleep and correctly identified as sleep by our feature extraction approach, false positive (FP) = VEEG depict awake and incorrectly identified as sleep by our feature extraction approach, true negative (TN) = VEEG depict awake and correctly as awake identified our feature extraction approach, false negative (FN) = VEEG depict sleep and incorrectly identified as awake by our feature extraction approach^

As proof of study, we have analyzed other neonatal facial color palettes extracted from Fluke^®^ SmartView. Additional file 3: Table S3 shows the statistical results achieved using multiple color palettes such as amber, high contrast, red-blue, hot metal, and grayscale. In contrast to the results shown in Table 1 Fluke^®^ multiple color palettes depict disproportionate results such as high contrast-AlexNet (FCL8), InceptionV3-Hot-metal (FCL), GoogLeNet-Grayscale(FCL), and VGG19-Red-Blue (FCL6) achieved the best values for Se are 84.8%, 76.3%, 73.0%, and 81.1% respectively. Similarly, VGG-19-Amber (FCL) shows the best values for Sp is 87.8%. However, overall, Ac obtained from these color palates are quite low; VGG19-High Contrast (FCL7) shows the best Ac of 65.6%. One of the main reason is that the range of these Fluke^®^ color palettes are quite narrow, as shown in Additional file 4: Figure S1.

In general, the statistical results of the pre-trained CNNs model as a feature extractor to classify neonatal sleep and wake states are quite modest [20]. One of the main reasons for attaining such modest accuracy is that all the existing pre-trained CNNs network were trained on natural images such as animals, flowers, sceneries, and automobiles, etc. The feature patterns of pre-trained CNNs networks classes are quite different from our neonate’s database, that makes it difficult for existing CNNs to classify neonate’s sleep and wake states [40, 41]. The motivation for using pre-trained CNNs as feature extraction is that it doesn’t demand a lot of computational capacity, and it is quite robust as we do not need to retain the network; these attributes compel us to start with feature extraction approach to classifying neonatal sleep and wake states. However, experimental analysis depicts that this approach doesn’t offer the promising results to act as an aided tool for clinicians to classify neonates’ sleep and wake states unobtrusively. Nevertheless, as there are no such studies has been reported in the literature by analyzing the neonatal facial videos to classify sleep and wake states using CNNs as feature extractor. This research could be helpful for future studies to adopt other techniques (e.g., transfer learning or dedicated CNNs) to classify neonatal sleep and wake states using video frames to achieve better accuracy.

### Conclusions

This work experimentally verified the achievability of unobtrusive neonatal sleep and wake states via automatic classification using a video frames from fluke^®^ camera. Five-fold cross-validation depicts the modest accuracy of 65.3% from pre-trained AlexNet at FCL7, compared with VEEG annotated data by a neurologist for sleep and wake states. In the future, the transfer learning approach/dedicated CNNs and more datasets collection with different ethnic groups will be the next step of our research work.

## Limitations of the study

It is also important to note that this is a preliminary study, where video data collection took place in a controlled environment with fixed camera placement, stable lighting conditions, and under the supervision of nurses and pediatricians. Furthermore, as for this proof-of-point study, we analyzed the variations in neonatal facial pattern no clear sleep-related issues (albeit with various reasons of hospital admission). The accuracy concerning to those with sleep syndromes have remained unclear. This article focus only on two-state (sleep and wake) classification, and the dedicated design of deep learning architecture to classify neonatal sleep stages is on the foremost next step of this research work.

## Supplementary information

**Additional file 1: Table S1.** Neonatal body condition before the collection of video and VEEG data, The detailed descriptions of the demographics and physical conditions of neonates.

**Additional file 2: Table S2.** Overall ConvNet’s architecture, The details descriptions of all the pre-trained model has been mentioned.

**Additional file 3: Table S3.** Neonatal sleep and wake states classification results using Fluke® multiple colors palattes, statistical results achieved using multiple color palettes such as amber, high contrast, red-blue, hot metal, and grayscale.

**Additional file 4: Figure S1. **Fluke® color palettes range.

## Data Availability

At this point the data used in this paper cannot be shared or released to a third party.

## Abbreviations

FCL’s: Fully-connected layers
VEEG: Video electroencephalogram
CNNs: Convolutional neural networks
PCA: Principal Component Analysis
SVM: Support vector machine
