# Supplementary material for: Can pre-trained convolutional neural networks be directly used as a feature extractor for video-based neonatal sleep and wake classification?
Source: BMC Res Notes. 2020 Nov 4;13:507. doi: 10.1186/s13104-020-05343-4 (PMC7641846; doi:10.1186/s13104-020-05343-4)
Supplement: Supplementary file 1 — Additional file 1: Table S1. Neonatal body condition before the collection of video and VEEG data, The detailed descriptions of the demographics and physical conditions of neonates. [file 13104_2020_5343_MOESM1_ESM.pdf]

Table S1 Neonatal body condition before the collection of video and VEEG data

#	Weight(kg)	GA(wk^{+d})	PMA(wk^{+d})	S/W	Reason for Admission
1	2.66	34	35+5	20/100	Preterm
2	4.53	39	42+4	20/100	Fever
3	3.36	39 +2	40+6	105/15	Cyanosis
4	2.91	37 +1	38+0	50/70	Jaundice
5	2.35	34 +6	35+3	60/60	emesis
6	2.83	38 +5	39+0	42/78	Hyoglycemia
7	3.22	40+5	41+1	95/25	Jaundice

S: sleep, W: wake, GA: gestational age, PMA: postmenstrual age during data collection, W= weight (kg)
