# Supplementary material for: Can pre-trained convolutional neural networks be directly used as a feature extractor for video-based neonatal sleep and wake classification?
Source: BMC Res Notes. 2020 Nov 4;13:507. doi: 10.1186/s13104-020-05343-4 (PMC7641846; doi:10.1186/s13104-020-05343-4)
Supplement: Supplementary file 2 — Additional file 2: Table S2. Overall ConvNet’s architecture, The details descriptions of all the pre-trained model has been mentioned. [file 13104_2020_5343_MOESM2_ESM.pdf]

Table S2 Overall ConvNet's architecture.

A	B	C	D	E	F
**VGG16**	**VGG19**	**AlexNet**	**GoogLeNet**	**Inception-V3**	**ResNet -18**
3x3Conv,64<br>3x3Conv,64	3x3Conv,64<br>3x3Conv,64	11x11Conv3	112x112Conv64	2x(149x149Conv2D32)<br>149x149Conv32(ReLu)<br>2x(147x147Conv2D32)<br>147x147Conv32(ReLu)<br>2x(147x147Conv2D 64)<br>147x147Conv64(ReLu)	7x7Conv,64
maxpool	maxpool	maxpool	maxpool(3x3/2)	maxpool(73x73x62)	
3x3Conv,128<br>3x3Conv,128	3x3Conv,128<br>3x3Conv,128	5x5Conv296	56x56Conv192	3x(73x73Conv2D80)<br>3x(71x71Conv2D192)	4(3x3Conv64)
maxpool	maxpool		maxpool(3x3/2))	maxpool(35x35x192)	maxpool
3x3<br>Conv,256<br>3x3Conv,256<br>3x3Conv,256	3x3Conv,256<br>3x3Conv,256<br>3x3Conv,256<br>3x3Conv,256	3x3Conv384<br>3x3Conv384<br>3x3Conv256	28x28Conv256<br>28x28Conv480	Inception1(35x35x256)<br>Inception2(35x35x288)<br>Inception3(35x35x288)<br>5x(Inception4(17x17x768))<br>Inception9(8x8x1280)<br>Inception10(8x8x2048)<br>Inception11(8x8x2048)	3x3Conv,128<br>3x3Conv,128<br>3x3Conv,128<br>3x3Conv,128
maxpool	maxpool	maxpool	maxpool(3x3/2))	globalmaxpool(1x1x2048)	maxpool
3x3Conv,512<br>3x3Conv,512<br>3x3Conv,512	3x3Conv,512<br>3x3Conv,512<br>3x3Conv,512<br>3x3Conv,512		3x(14x14Conv512)<br>14x14 Conv 528<br>14x14Conv32		3x3Conv,256<br>3x3Conv,256<br>3x3Conv,256<br>3x3Conv,256
maxpool	maxpool		maxpool(3x3/2))		maxpool
3x3Conv,512<br>3x3Conv,512<br>3x3Conv,512	3x3Conv,512<br>3x3Conv,512<br>3x3Conv,512<br>3x3Conv,512		7x7Conv832<br>7x7Conv1024		3x3Conv,512<br>3x3Conv,512<br>3x3Conv,512<br>3x3Conv,512
maxpool	maxpool	maxpool	avgpool(7x7/1)		
**FCL6-4096**	**FCL6-4096**	**FCL6-2048**	1x1Conv1024	FCL1008	
**FCL7-4096**	**FCL7-4096**	**FCL7-2048**	dropout(40%)		
**FCL8-1000**	**FCL8-100**	**FCL8-1000**	**FCL-1000**	**FCL-1000**	**FCL-1000**
softmax	softmax	softmax	softmax	softmax	softmax
