# Supplementary material for: Can pre-trained convolutional neural networks be directly used as a feature extractor for video-based neonatal sleep and wake classification?
Source: BMC Res Notes. 2020 Nov 4;13:507. doi: 10.1186/s13104-020-05343-4 (PMC7641846; doi:10.1186/s13104-020-05343-4)
Supplement: Supplementary file 3 — Additional file 3: Table S3. Neonatal sleep and wake states classification results using Fluke® multiple colors palattes, statistical results achieved using multiple color palettes such as amber, high contrast, red-blue, hot metal, and grayscale. [file 13104_2020_5343_MOESM3_ESM.pdf]

Table S3. Neonatal sleep and wake states classification results using Fluke® multiple colors palattes

Video Frame			Se%	Sp%	Ac%	P%
			SVM			
AlexNet	High Contrast	FCL6	64.8	57.8	63.9	65.2
		FCL7	64.8	55.8	62.7	64.2
		FCL8	71.0	54.5	62.4	65.6
VGG-16		FCL6	83.2	49.0	63.2	66.6
		FCL7	80.7	46.5	61.2	64.8
		FCL8	73.5	50.9	64.1	64.6
VGG-19		FCL6	81.2	51.8	65.4	67.3
		FCL7	83.2	51.5	**65.6**	67.7
		FCL8	**84.8**	43.9	56.9	64.5
Resnet-18		FCL	70.9	47.5	59.2	62.3
InceptionV3		FCL	80.1	47.8	56.8	65.2
GoogLeNet		FCL	72.4	50.3	59.8	64.0
AlexNet	Hot metal	FCL6	56.5	59.3	57.4	62.9
		FCL7	62.4	54.8	58.7	62.8
		FCL8	62.7	50.0	56.3	61.3
VGG-16		FCL6	51.5	61.7	55.7	62.2
		FCL7	51.8	60.4	54.9	61.5
		FCL8	51.8	60.4	56.1	61.5
VGG-19		FCL6	69.7	48.4	57.4	62.3
		FCL7	73.6	46.1	58.1	62.5
		FCL8	71.5	43.9	59.1	60.9
Resnet-18		FCL	70.9	47.5	59.2	62.3
InceptionV3		FCL	**76.3**	48.4	63.5	64.4
GoogLeNet		FCL	70.1	51.8	60.3	64.0
AlexNet	Red-blue	FCL6	77.2	45.0	62.3	63.2
		FCL7	77.6	45.8	62.1	63.6
		FCL8	65.9	54.1	61.3	63.7
VGG-16		FCL6	72.9	48.4	59.9	63.3
		FCL7	63.6	51.0	56.8	61.3
		FCL8	64.8	52.4	57.7	62.4
VGG-19		FCL6	**81.1**	44.5	64.3	64.1
		FCL7	79.1	42.3	61.5	62.6
		FCL8	73.4	44.9	62.5	61.9
Resnet-18		AFCL	59.1	63.4	61.3	66.4
InceptionV3		AFCL	67.2	51.3	56.3	62.8
GoogLeNet		AFCL	78.4	44.6	59.4	63.5
AlexNet	Amber	FCL6	37.4	78.4	52.4	67.8
		FCL7	49.5	69.7	58.5	66.3
		FCL8	49.5	69.7	58.9	66.3
VGG-16		FCL6	44.4	70.7	57.6	64.7
		FCL7	41.2	74.4	59.9	66.0
		FCL8	45.9	67.1	57.6	62.7
VGG-19		FCL6	35.9	**87.8**	60.2	78.0
		FCL7	33.2	81.7	58.2	68.7
		FCL8	30.9	83.5	52.6	69.4
Resnet-18		FCL	42.4	75.5	58.9	67.6

InceptionV3		FCL	59.0	58.4	59.1	63.1
GoogLeNet		FCL	48.3	63.1	55.9	61.2
AlexNet	Grayscale	FCL6	53.4	58.4	55.3	61.1
		FCL7	56.9	56.9	56.8	61.7
		FCL8	60.1	55.6	58.3	62.3
VGG-16		FCL6	45.4	71.7	56.6	63.7
		FCL7	41.2	73.4	58.9	65.0
		FCL8	50.9	68.1	58.6	63.7
VGG-19		FCL6	51.9	64.6	56.7	64.2
		FCL7	57.3	57.6	57.1	62.1
		FCL8	61.7	55.3	57.9	62.2
Resnet-18		FCL	66.9	48.5	60.2	61.4
InceptionV3		FCL	70.4	45.9	59.1	61.4
GoogLeNet		FCL	**73.0**	43.0	57.3	61.0
