# Supplementary figures and images for: Can pre-trained convolutional neural networks be directly used as a feature extractor for video-based neonatal sleep and wake classification?
Source: BMC Res Notes. 2020 Nov 4;13:507. doi: 10.1186/s13104-020-05343-4 (PMC7641846; doi:10.1186/s13104-020-05343-4)

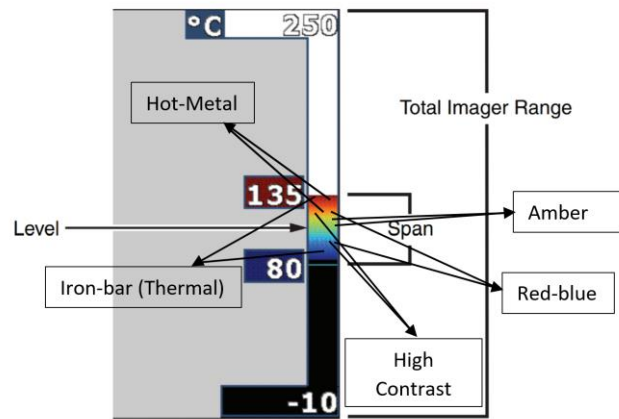

Figure S3 Fluke® multiple palette color range .

Supplement: Supplementary file 4 — Additional file 4: Figure S1. Fluke® color palettes range. [file 13104_2020_5343_MOESM4_ESM.pdf]
